# Serum total cholesterol, calcium, and IgG as discriminatory markers of lupus nephritis in children with systemic lupus erythematosus

**DOI:** 10.3389/fped.2026.1889717

**Published:** 2026-08-03

**Authors:** Jun Jiang, Qing He, Huixin Gao, Wenzheng Li, Hao Zheng

**Affiliations:** Department of Clinical Laboratory, Guangzhou Women and Children’s Medical Center, Guangzhou Medical University, Guangzhou, Guangdong Province, China

**Keywords:** childhood-onset systemic lupus erythematosus, immunoglobulin G, lupus nephritis, total calcium, total cholesterol

## Abstract

**Objective:**

Childhood-onset systemic lupus erythematosus (cSLE) commonly involves lupus nephritis (LN), a severe complication. Reliable serological discriminatory markers for LN are lacking. This study aimed to identify routine laboratory parameters associated with prevalent LN in cSLE.

**Methods:**

A total of 187 children with diagnosed SLE were divided into LN group (*n* = 84) and non-LN groups (*n* = 103) based on renal biopsy findings. Clinical and laboratory data were compared. Multivariate logistic regression identified independent associated factors for LN. Furthermore, the correlations between these laboratory parameters and renal function indicators were analyzed. Receiver operating characteristic (ROC) curves were plotted to evaluate the discriminatory performance of these indicators for LN.

**Results:**

Compared to the non-LN group, the LN group showed significant alterations in multiple serum parameters (e.g., lipids, coagulation markers, immunoglobulins, complement). Multivariate logistic regression analysis identified decreased serum total calcium (Ca) and IgG, and elevated serum total cholesterol (TC) as independent associated factors for LN (all *P* < 0.05). Serum TC correlated positively, and serum total calcium correlated negatively, with serum creatinine and 24-hour urine protein (all *P* < 0.05). The areas under the ROC curve (AUC) for discriminating the presence of LN using serum TC, Ca, and IgG alone were 0.762, 0.697, and 0.684, respectively. Their combination achieved the highest AUC of 0.852 (*95% CI*: 0.796∼0.908).

**Conclusion:**

A multivariate logistic regression model incorporating serum TC, Ca, and IgG levels demonstrates strong discriminatory utility for prevalent LN in children with SLE.

## 1 Introduction

Childhood-onset systemic lupus erythematosus (cSLE) is an autoimmune disease characterized by multisystem and multiorgan involvement. It is typically defined as SLE diagnosed before the age of 18 years, accounting for approximately 10%–20% of all SLE cases and 15%–25% of pediatric rheumatic diseases (1, 2). Compared to adult-onset SLE, cSLE is characterized by more acute onset, more pronounced systemic symptoms, higher disease activity, more extensive and severe organ involvement (particularly in critical organs such as the kidneys), and a poorer prognosis (3). Lupus nephritis is one of the most common and severe complications of cSLE. More than half of affected children present with renal involvement at the time of SLE diagnosis, with proliferative nephritis (such as Class IV) being the most common pathological subtype (4, 5). As the disease progresses or relapses, a proportion of children with LN may progress to chronic kidney disease or even end-stage renal failure. Therefore, early recognition and timely intervention for LN are crucial and can significantly improve the prognosis for these children.

Currently, the clinical monitoring of LN primarily relies on tracking renal function parameters—such as serum creatinine, urinalysis, urine protein-to-creatinine ratio, and 24-hour urine protein quantification—and immunological markers such as anti-dsDNA antibodies and complement levels (C3, C4). However, renal biopsy remains the gold standard for definitive diagnosis and pathological classification of LN. Studies have revealed discrepancies between these conventional laboratory markers and histopathological findings, indicating that proteinuria and renal function indicators alone cannot accurately reflect the true state of renal activity (6, 7). Furthermore, the invasive nature of renal biopsy makes it unsuitable for frequent, repeated dynamic monitoring, limiting its application in cSLE patients. In recent years, many studies have identified various novel urinary biomarkers with considerable potential for diagnosing and monitoring LN (8, 9). However, compared to serum samples, urine is subject to more interfering factors, is more difficult to preserve, involves higher testing costs, and lacks standardized methods. Consequently, these biomarkers remain in the research phase and have not yet been routinely implemented in clinical practice (10, 11). Therefore, there is a need to identify widely available, technically stable, and clinically established serological markers to assist clinicians in rapidly and conveniently assessing the condition of cSLE.

In clinical practice, the value of certain routinely available serum parameters for LN may not yet be fully explored. Therefore, this study aims to analyze and compare the profiles of common laboratory parameters between cSLE with and without LN, in order to further investigate the relationship of these indicators with the development and progression of LN and to explore their potential clinical applicability, thereby providing a reference for clinical diagnosis and treatment.

## 2 Materials and methods

### 2.1 Study population

This retrospective study enrolled 187 children diagnosed with SLE at the Women and Children's Medical Center affiliated with Guangzhou Medical University between January 2023 and December 2025. The cohort included 49 males and 138 females, aged 4 to 16 years (mean age: 11 years). All patients met the 2019 European League Against Rheumatism/American College of Rheumatology (EULAR/ACR) classification criteria for SLE (12). The exclusion criteria were as follows: (1) Concurrent infections; (2) Malignancies; (3) Hematological disorders; (4) Lymphoproliferative diseases; (5) Other autoimmune diseases; (6) Changes in treatment regimen within the past 3 months; (7) Receipt of intravenous immunoglobulin or biological agents within the previous 6 months; (8) Incomplete clinical data.

The cSLE patients were divided into lupus nephritis (LN, *n* = 84) and non-lupus nephritis (non-LN, *n* = 103) groups according to renal histopathological findings. All LN patients were proven by renal biopsy according to the 2018 Revision of the International Society of Nephrology/Renal Pathology Society (ISN/RPS) classification for lupus nephritis (13). The baseline clinical characteristics of all participants are shown in Table 1. The two groups were comparable in terms of age, gender, and disease duration, with no statistically significant differences. The study was conducted in accordance with the Declaration of Helsinki and approved by the Ethics Committee of Guangzhou Women and Children's Medical Center (Approval No. 2023-318B01).

**Table 1:** The basic clinical characteristics of SLE patients.

Parameters	LN group (*n* = 84)	non-LN group (*n* = 103)	*P* value
Female, n(%)	57 (67.86%)	81 (78.64%)	0.095
Age (year)	11.29 ± 2.25	10.78 ± 2.34	0.134
Duration (month)	3 (1,12)	6 (2,12)	0.340
SLEDAI-2000 (score)	10 (8,14)	6 (2,11)	**<0**.**001**
ISN/RPS Class of lupus nephritis			
Class I + II + III	28 (33.34%)	/	
Class IV	31 (36.90%)	/	
Class V	25 (29.76%)	/	

Data are presented as mean ± standard deviation (SD) for normally distributed continuous variables, median [interquartile range (IQR)] for non-normally distributed continuous variables and categorical variables as n (%). Bold values indicate statistical significance (*P*<0.05). SLE, Systemic lupus erythematosus; LN, Lupus nephritis; SLEDAI-2000, Systemic Lupus Erythematosus Disease Activity Index 2000; ISN/RPS, International Society of Nephrology/Renal Pathology Society.

### 2.2 Data collection and laboratory measurements

Clinical characteristics, demographic data, and laboratory test results for the enrolled children with SLE were collected retrospectively from the hospital's Health Information System (HIS) and the Laboratory Information System (LIS). All laboratory data were obtained at the time of admission, prior to any adjustment of the patients’ medication regimens. Routine laboratory parameters, including complete blood counts, inflammatory markers, routine biochemical tests, and immunological assays, were extracted for analysis. The specific methodologies and instruments were as follows:

C-reactive protein (CRP) was quantified using an Ottoman-1000 Specific Protein Analyzer (Ottoman, China) with its corresponding reagents. Erythrocyte sedimentation rate (ESR) was determined using a Test1 THL Automated Erythrocyte Sedimentation Rate Analyzer (ALIFAX, Italy). Routine hematology parameters, including hemoglobin quantification (Hb) and platelet count (PLT), are measured using the XN-2000 Automated Hematology Analyzer (Sysmex, Japan) and corresponding reagents. Biochemical Parameters including Alanine aminotransferase (ALT), Albumin (ALB), Albumin/globulin ratio (ALB/GLO), Lactate dehydrogenase (LDH), Creatine kinase (CK), Creatinine (Cr), 24-hour urinary protein quantification (UPRO), total Calcium (Ca), Phosphorus (P), Total cholesterol (TC), Triglycerides (TG), High-density lipoprotein cholesterol (HDL-C), and Low-density lipoprotein cholesterol (LDL-C) were measured using a HITACHI LABOSPECT 008AS automated biochemical analyzer (Hitachi, Japan) with compatible reagents (Medicalsystem, China). The albumin-adjusted Calcium (aCa) was calculated using the Payne formulas (14), and estimated glomerular filtration rate (eGFR) was calculated using the Schwartz Formula (2009) (15). Coagulation Parameters including Prothrombin time (PT), PT activity (PTA), International normalized ratio (INR), Activated partial thromboplastin time (APTT), Thrombin time (TT), Fibrinogen (FIB), and D-dimer (DD) were assayed using a STA-R MAX automated coagulation analyzer (Stago, France) and corresponding reagents. Immunological Parameters, Immunoglobulin (IgG, IgM, IgA) and complement (C3, C4) levels were measured using an Immage 800 Immunochemistry System (Beckman Coulter, USA) and corresponding reagents.

### 2.3 Statistical analysis

Statistical analyses were performed using SPSS Statistics 23.0, GraphPad Prism 9.0, and MedCalc 23.1.6 software. Continuous data were assessed for normality using the Kolmogorov–Smirnov test. Normally distributed data are expressed as the mean ± standard deviation, and were compared between groups using the independent samples t-test; correlations were analyzed using Pearson's correlation coefficient. Data not following a normal distribution are expressed as median (interquartile range); comparisons between groups were performed using the Mann–Whitney U test, and correlation analysis was performed using Spearman's correlation coefficient. In constructing the logistic regression model, variables that affected the grouping of the dependent variable were first excluded. Indicators showing statistically significant differences between the two groups (*P* < 0.05) were then identified and entered into univariate binary logistic regression analysis to select candidate variables (*P* < 0.05). Subsequently, multicollinearity diagnostics were performed, and variables with a variance inflation factor (VIF) > 10 were excluded. We then selected the variable with the largest univariate effect as the representative variable and performed a multivariate binary logistic regression analysis using it along with the remaining candidate variables for which VIF was less than 10, employing forward stepwise selection (Wald) in SPSS (Default settings: ENTRY = 0.05; REMOVAL = 0.10). The final calculation results was visualized using a forest plot. Receiver operating characteristic (ROC) curves were used to evaluate the ability of these laboratory indicators to discriminate prevalent lupus nephritis. A two-tailed P-value of less than 0.05 was considered statistically significant.

## 3 Results

### 3.1 Laboratory parameters in cSLE with and without lupus nephritis

A comparison of routine laboratory data between children with lupus nephritis (LN group) and those without (non-LN group) is presented in Table 2. Compared to the non-LN group, patients in the LN group showed significantly elevated levels of serum Creatinine (Cr), estimated Glomerular filtration rate (eGFR), 24-hour urinary protein quantification (UPRO), Total Cholesterol (TC), Triglycerides (TG), High-density lipoprotein cholesterol (HDL-C), Low-density lipoprotein cholesterol (LDL-C), Prothrombin time activity (PTA), Fibrinogen (FIB), D-dimer (DD), and Immunoglobulin M (IgM). Conversely, levels of alanine Aminotransferase (ALT), Albumin (ALB), Albumin-to-globulin ratio (ALB/GLO), total Calcium (Ca), Phosphorus (P), Prothrombin time (PT), International normalized ratio (INR), Activated partial thromboplastin time (APTT), Immunoglobulin G (IgG), and Complement 3 (C3) were significantly lower in the LN group. All these differences were statistically significant (*P* < 0.05).

**Table 2:** Comparison of routine laboratory parameters between the LN group and the Non-LN group.

Parameters	LN group (*n* = 84)	Non-LN group (*n* = 103)	*P* value
CRP(mg/L)	0.51 (0.47,1.23)	0.52 (0.49,1.15)	0.448
ESR(mm/h)	19.00 (9.00,31.75)	15.00 (7.00,29.00)	0.303
Hb(g/L)	112.89 ± 20.82	115.67 ± 19.53	0.349
PLT(10^9^/L)	295.38 ± 106.99	286.18 ± 104.03	0.553
ALT(U/L)	16.50 (11.25,27.00)	20.00 (13.00,37.00)	**0**.**041**
ALB(g/L)	38.60 (30.95,44.60)	42.80 (39.40,45.20)	**0**.**004**
ALB/GLO	1.46 ± 0.46	1.61 ± 0.51	**0**.**043**
LDH(U/L)	231.50 (201.25,287.75)	215.00 (187.00,321.00)	0.482
CK(U/L)	29.00 (19.00,40.00)	32.00 (23.00,54.00)	0.100
Cr(*μ*mol/L)	48.00 (37.25,60.50)	39.00 (32.00,46.00)	**<0**.**001**
eGFR(mL/min/1.73m^2^)	111.45 ± 36.95	135.50 ± 28.49	**<0**.**001**
UPRO(g/24 h)	1.37 (0.25,4.15)	0.09 (0.06,0.24)	**<0**.**001**
Ca(mmol/L)	2.20 (2.07,2.32)	2.33 (2.22,2.41)	**<0**.**001**
aCa(mmol/L)	2.29 (2.12,2.37)	2.28 (2.22,2.33)	0.809
P(mmol/L)	1.30 (1.29,1.38)	1.39 (1.26,1.61)	**0**.**027**
TC(mmol/L)	6.28 ± 2.45	4.45 ± 1.33	**<0**.**001**
TG(mmol/L)	1.91 (1.39,2.99)	1.33 (0.96,2.13)	**<0**.**001**
HDL-C(mmol/L)	1.70 ± 0.76	1.29 ± 0.52	**<0**.**001**
LDL-C(mmol/L)	3.85 ± 1.94	2.67 ± 1.05	**<0**.**001**
PT(s)	12.58 ± 0.74	12.90 ± 0.84	**0**.**007**
PTA-%	111.50 (104.25,120.00)	107.00 (95.00,118.00)	**0**.**015**
INR	0.94 (0.91,0.98)	0.96 (0.91,1.03)	**0**.**016**
APTT(s)	34.45 (31.18,36.85)	36.60 (33.80,40.90)	**0**.**001**
TT(s)	18.10 (17.20,18.98)	18.20 (17.60,19.00)	0.350
FIB(g/L)	2.97 (2.51,3.60)	2.75 (2.37,3.18)	**0**.**023**
D-D(mg/L)	0.40 (0.22,1.27)	0.25 (0.22,0.62)	**0**.**004**
IgG(g/L)	9.22 ± 3.94	12.34 ± 4.98	**<0**.**001**
IgA(g/L)	1.82 ± 0.73	1.76 ± 0.72	0.554
IgM(g/L)	0.98 (0.72,1.47)	0.84 (0.62,1.25)	**0**.**027**
C3(g/L)	0.62 ± 0.25	0.71 ± 0.29	**0**.**035**
C4(g/L)	0.12 ± 0.08	0.13 ± 0.07	0.215

Data are presented as mean ± standard deviation (SD) for normally distributed continuous variables, and median [interquartile range (IQR)] for non-normally distributed continuous variables. Bold values indicate statistical significance (*P*<0.05). CRP, C-reactive protein; ESR, Erythrocyte sedimentation rate; Hb, Hemoglobin quantification; PLT, Platelet count; ALT, Alanine aminotransferase; ALB, Albumin; ALB/GLO, Albumin/globulin ratio; LDH, Lactate dehydrogenase; CK, Creatine kinase; Cr, Creatinine; UPRO, 24-hour urinary protein quantification; Ca, total Calcium; aCa, albumin-adjusted Calcium; P, Phosphorus; TC, Total cholesterol; TG, Triglycerides; HDL-C, High-density lipoprotein cholesterol; LDL-C, Low-density lipoprotein cholesterol; eGFR, estimated glomerular filtration rate; PT, Prothrombin time; PTA, PT activity; INR, International normalized ratio; APTT, Activated partial thromboplastin time; TT, Thrombin time; FIB, Fibrinogen; DD, D-dimer; IgG, Immunoglobulin G; IgM, Immunoglobulin M; IgA, Immunoglobulin A; C3, complement 3; C4, complement 4.

### 3.2 Logistic regression analysis of associated factors for lupus nephritis

**Figure 1:**
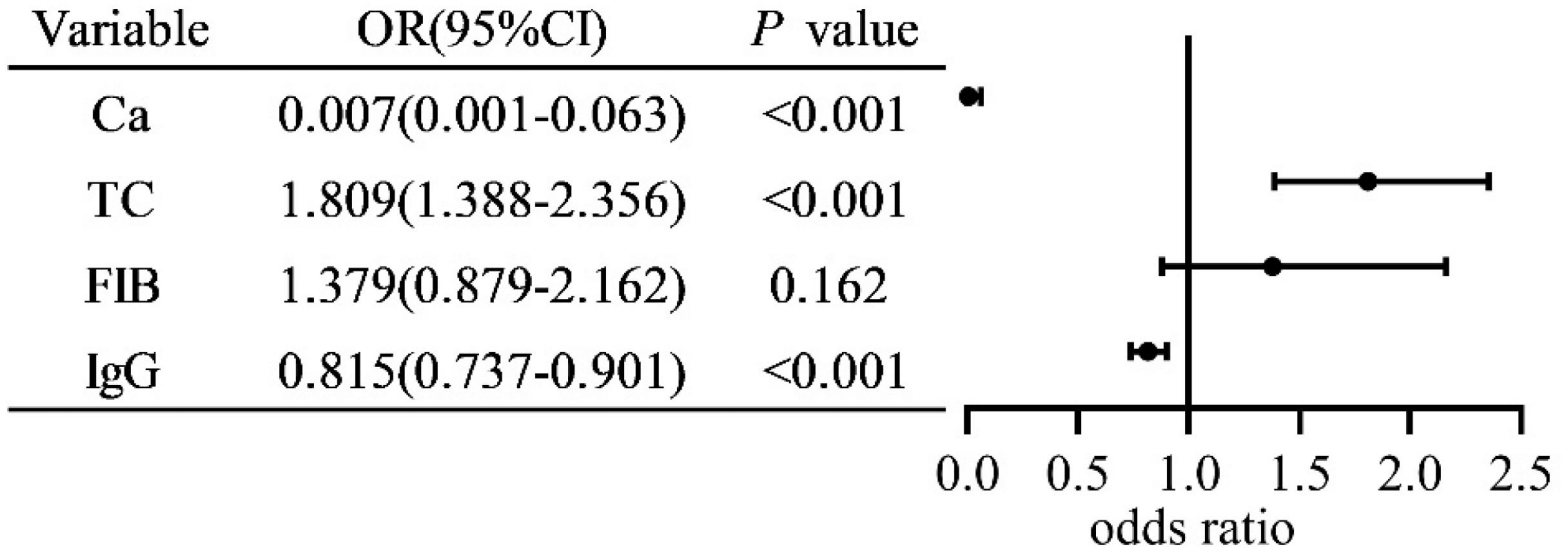
Forest plot of multivariable logistic regression analysis of lupus nephritis in cSLE. The plot visualizes the automated output results from a multivariable logistic regression model. Each variable is listed on the *Y*-axis, with its odds ratio (OR) and 95% confidence interval (CI) displayed on the *X*-axis on a logarithmic scale. The circle marker represents the point estimate of the OR. The horizontal line extending from the circle represents the 95% CI. The vertical dashed line at OR = 1 indicates the line of no effect. FIB whose 95% CI crosses this line are not statistically significant at the *P* < 0.05 level. OR >1 indicates an associated factor; OR <1 indicates a protective factor. Ca, total Calcium; TC, total cholesterol; FIB, fibrinogen; IgG, Immunoglobulin G.

Univariate logistic regression analysis was performed on laboratory parameters with significant between-group differences to identify candidate variables (*P* < 0.05). After excluding variables with VIF > 10 due to multicollinearity, TC and PT were retained as representative indicators. We then adjusted for factors influencing LN subgroup classification (SLEDAI-2 K, Cr, eGFR, and UPRO), and included the following candidate indicators—ALB/GLO, Ca, P, TC, TG, HDL, PT, APTT, FIB, IgG, IgM, and C3—as independent variables in a multivariate logistic regression model, with LN presence (1 = present, 0 = absent) as the dependent variable. The results of the regression model calculations were shown in Figure 1. Although FIB was automatically retained in the last iteration of the SPSS stepwise algorithm, its Wald test was not significant (*P* = 0.162). Therefore, FIB was not considered an independent associated factor. The final interpretable model was restricted to TC, Ca, and IgG, all of which showed *P* < 0.001. Accordingly, we focused our subsequent analysis and discussion on these three significant factors. These findings indicate that in children with SLE, decreased serum Ca and IgG levels, as well as elevated serum TC levels, were identified as potential associated factors for LN in cSLE.

### 3.3 Correlation of serum TC, Ca and IgG levels with renal function

**Figure 2:**
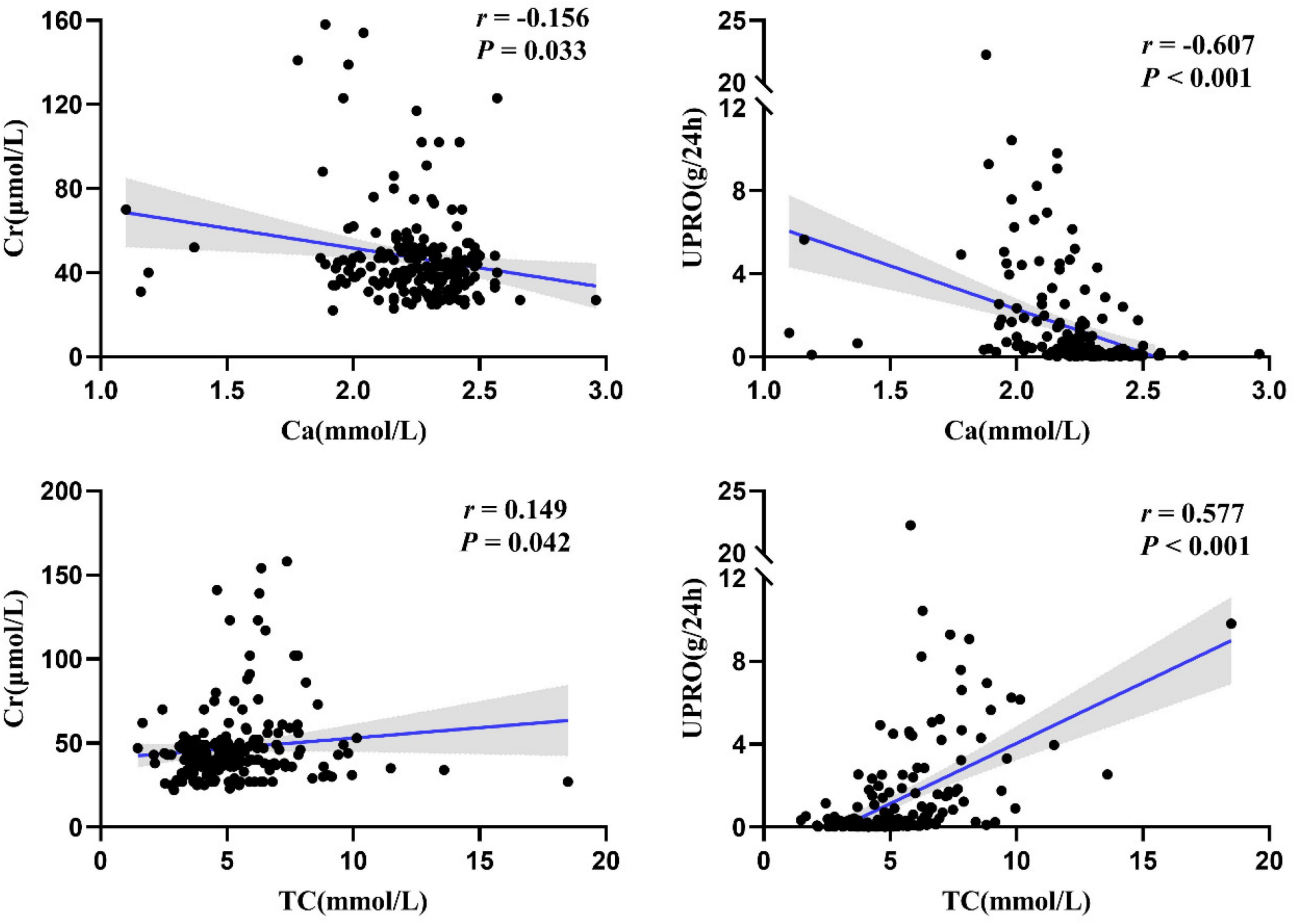
Correlations of serum total calcium and total cholesterol with renal function parameters. Four scatter plots sequentially illustrate the correlations of serum Ca with serum Cr and UPRO, as well as the correlations of serum TC with serum Cr and UPRO. On the right side of each scatter plot, the Spearman correlation coefficient **(*r*)** and the corresponding *P* value are displayed. *P* value <0.05 was considered statistically significant. Ca, total Calcium; Cr, creatinine; UPRO, 24 h urinary protein quantification; TC, total cholesterol.

Correlation analysis was performed to assess the relationships between the three indicators and renal function parameters. As shown in Figure 2, serum TC was positively correlated with serum Cr (r = 0.149, *P* < 0.05) and 24-hour urine protein quantification (r = 0.588, *P* < 0.05). In contrast, serum Ca was negatively correlated with serum Cr (r = −0.156, *P* < 0.05) and UPRO (r = −0.607, *P* < 0.05). However, serum IgG showed no significant correlation with serum Cr or UPRO.

### 3.4 ROC curve analysis of serum TC, Ca, and IgG for discriminating prevalent lupus nephritis

**Figure 3:**
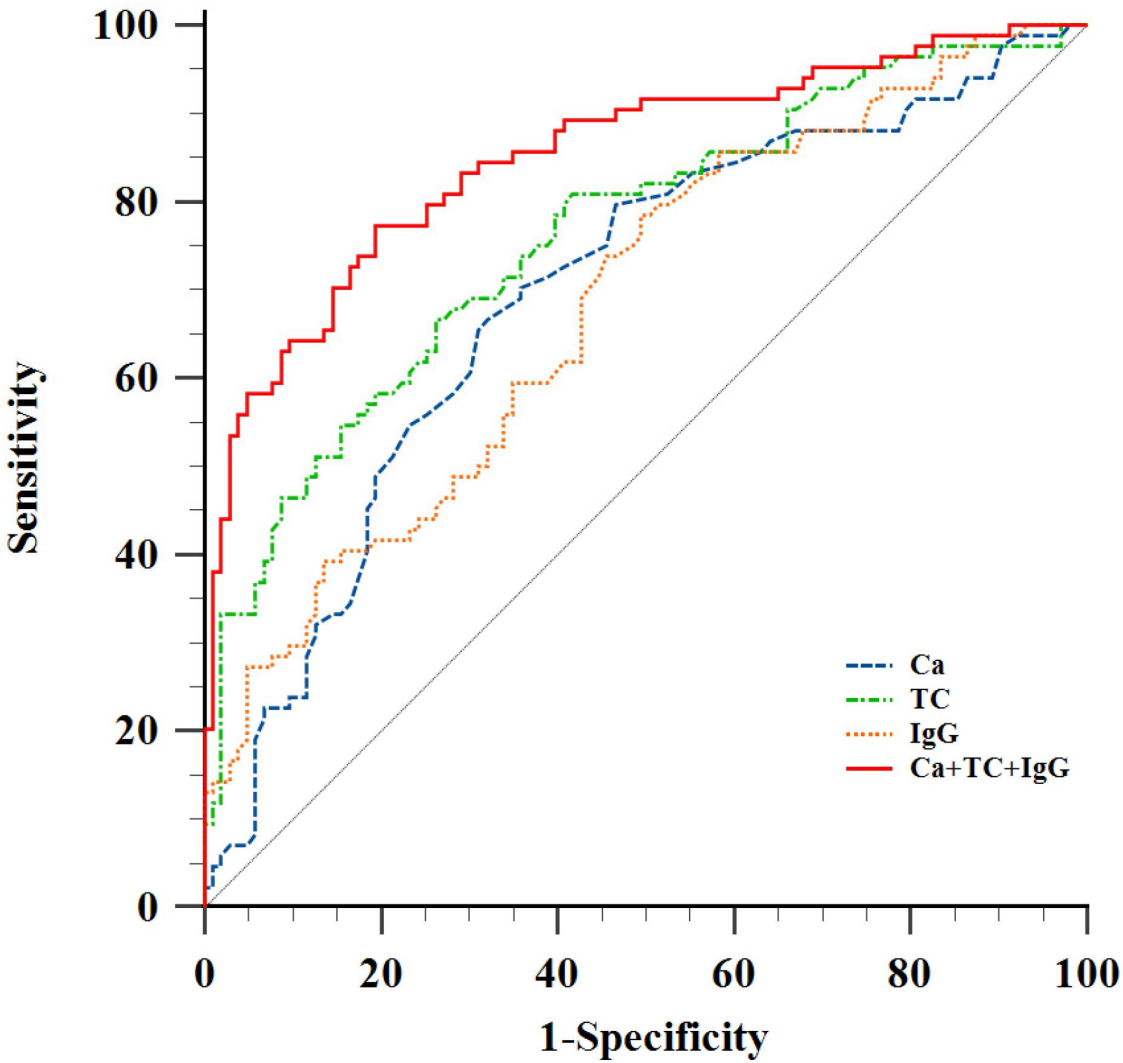
ROC curve analysis of single and combined indicators for discriminating lupus nephritis in cSLE. The ROC curves for serum TC, Ca, and IgG used to distinguish SLE children with versus without lupus nephritis are represented by blue, green, and orange dashed lines, respectively. The ROC curve for the combination of these three indicators is shown as a red solid line. Ca, total Calcium; TC, total cholesterol; IgG, Immunoglobulin G.

The discriminatory performance of serum TC, Ca, and IgG for LN was evaluated using ROC curve analysis. Individually, each indicators showed discriminative capacity for LN in children with SLE, with areas under the ROC curve (AUC) of 0.762 (*95% CI*: 0.693∼0.831) for TC, 0.697 (*95% CI*: 0.620∼0.773) for Ca, and 0.684 (*95% CI*: 0.608∼0.760) for IgG. The discriminative ability was highest when the three indicators were combined, yielding an AUC of 0.852 (*95% CI*: 0.796∼0.908), with sensitivity and specificity of 77.38% and 80.58%, respectively (Figure 3), indicating promising clinical applicability for identifying prevalent LN.

## 4 Discussion

Lupus nephritis (LN) is a leading cause of mortality in children with childhood-onset systemic lupus erythematosus (cSLE). Renal involvement in most pediatric patients occurs concurrently with or within two years of the onset of extra-renal manifestations (16). Clinical presentations of pediatric LN, such as asymptomatic proteinuria, hematuria, abnormal urine output, and edema, are often more acute and severe than in adults (17). Therefore, timely identification of LN is crucial for guiding appropriate treatment. Although renal biopsy remains the gold standard for diagnosing LN, its invasive nature, low patient acceptability, and unsuitability for repeated assessments limit its utility in pediatric patients. In recent years, numerous novel urinary biomarkers, such as activated leukocyte adhesion molecule (ALCAM), vascular cell adhesion molecule 1 (VCAM-1), neutrophil gelatinase-associated lipocalin (NGAL), soluble CD163 (sCD163), and monocyte chemotactic protein-1 (MCP-1),have shown promising diagnostic and monitoring value for LN in SLE (18–20). However, most of these emerging biomarkers are still in the research phase, with unstandardized testing methods and high costs, and pre-analytical variability, hindering their routine clinical application. Therefore, identifying economical, convenient, and reproducible serological markers holds significant clinical value. In this study, we retrospectively analyzed the routine serum profiles of cSLE and found that serum Total cholesterol (TC), Total Calcium (Ca), and Immunoglobulin G (IgG) as biomarkers significantly associated with LN. We further explored the discriminatory power of these indicators, both individually and in combination, in the timely identification of LN.

A growing body of evidence suggests dyslipidemia may play a significant role in the pathogenesis of LN. Studies indicate that approximately 50%–70% of patients with LN exhibit lipid metabolism disorders (21), with hypercholesterolemia being associated with the degree of proteinuria (22), which significantly influencing the long-term prognosis of LN. In this study, analysis of lipid metabolism parameters in cSLE revealed significantly higher serum levels of TC, TG, HDL-C, and LDL-C in the LN group compared to the non-LN group. These findings are consistent with reports by Liu et al. (23) and Huang et al. (24), suggesting a high prevalence of dyslipidemia in LN patients. Our multivariate logistic regression analysis further identified elevated TC as an independent factor closely associated with the development of LN. TC levels showed a moderate positive correlation with 24-hour urinary protein quantification and a certain degree of correlation with serum Cr, aligning with the findings of Feng et al. (25) Collectively, these results suggest that hypercholesterolemia is associated with proteinuria severity in LN, and TC may serve as an independent associated factor for renal involvement in cSLE. SLE is a chronic inflammatory disease in which elevated inflammatory cytokines can disrupt normal lipoprotein metabolism, contributing to dyslipidemia. In LN patients, significant proteinuria and subsequent hypoalbuminemia may trigger a compensatory increase in hepatic lipoprotein synthesis, further exacerbating lipid abnormalities. Additionally, lipid metabolites may exacerbate lupus-associated autoimmune responses and renal inflammation through immune mechanisms (26, 27). Therefore, a bidirectional, mutually reinforcing relationship likely exists between hypercholesterolemia and the progression of lupus nephritis.

Hypocalcemia may be both a consequence and a biomarker of disease activity in LN. Several clinical studies have confirmed that decreased serum calcium levels are closely associated with the activity of SLE and LN (28, 29). In this study, children with LN exhibited significantly lower serum total calcium than the non-LN group, whereas albumin-corrected calcium levels were comparable between the two groups, suggesting that ionized calcium remains relatively stable in LN patients. The reduction in total calcium is likely attributable to hypoalbuminemia secondary to massive proteinuria, which promotes urinary loss of calcium-binding proteins and consequently lowers the protein-bound calcium fraction. Meanwhile, declining renal function may impair active vitamin D synthesis and intestinal calcium absorption, potentially contributing to decreased total calcium. This interpretation is reinforced by the negative correlations we observed between serum calcium and both serum creatinine and 24-hour urinary protein quantification. Some studies have linked hypocalcemia to an enhanced cellular immune state in SLE and proposed that inflammatory cytokines may suppress parathyroid hormone activity, thus affecting calcium metabolism (30). However, our data did not reveal evidence of overt calcium homeostasis disruption. In addition, recent evidence suggested that uncorrected total calcium may actually be the best and most practical surrogate marker for ionized calcium, raising questions about the reliability and sensitivity of corrected calcium formulas (31, 32). Nevertheless, the actual ionized calcium status in children with LN needs further investigation. In this study, we focused on total calcium—a clinically meaningful, easily accessible, and requires no additional calculation—for discussion and analysis. Based on these findings, it is suggested that monitoring and appropriately correcting serum total calcium levels represent an important component of clinical management in LN.

Contrary to the classical paradigm of SLE-associated polyclonal B-cell hyperactivation and hypergammaglobulinemia, our study revealed that serum IgG levels were significantly lower in children with LN compared to the non-LN group, and that low IgG emerged as an independent associated factor for prevalent LN. Possible causes for seemingly paradoxical finding as follows: In active LN, particularly those presenting with nephrotic syndrome, the damaged glomerular filtration barrier may permit substantial urinary loss of IgG due to its relatively low molecular weight (approximately 150 kDa), potentially offsetting the enhanced synthesis driven by B-cell hyperactivation. However, this hypothesis is not directly supported by our data, as we observed no significant correlation between serum IgG levels and 24-hour urine protein quantification or creatinine—a discordance that may reflect the inherent limitations of a retrospective cross-sectional design. Alternatively, prior studies have shown that low levels of certain IgG subtypes (such as IgG2) are associated with SLE disease activity (33, 34), suggesting that subclass-specific alterations may be more relevant than total IgG concentration alone. Nonetheless, the specific mechanisms underlying this inverse association remain incompletely understood and require further research. Clinically, while serum IgG monitoring in LN patients may offer useful adjunctive information, its interpretation should not be based on isolated measurements but rather on dynamic trends evaluated in conjunction with other laboratory parameters for a comprehensive assessment.

The combination of serum TC, Ca, and IgG emerged as the most effective predictor of LN in our study, achieving an AUC of 0.852 with 77.38% sensitivity and 80.58% specificity, outperforming any single indicator. As these parameters are routinely measured in clinical practice, they offer the advantages of rapid obtained, low cost, and suitability for repeated monitoring. This accessible biomarker serves as a valuable supplement to traditional serological indicators, providing clinicians with a convenient and evidence-based reference for discriminating prevalent LN in children with SLE. Notably, our study is exploratory and complementary, aiming to underscore the discriminatory signals from routinely available parameters rather than to replace traditional immunological assessments.

This study has several limitations. First, as a retrospective analysis, the findings may be influenced by selection bias. Furthermore, in real-world studies, we cannot completely rule out residual confounding factors associated with long-term immunomodulatory therapy. Thus, our findings reflect predictive indicators under therapeutic conditions rather than innate pathophysiological predictors at baseline. Prospective validation in a cohort of newly diagnosed cases is needed in the future. Second, because these indicators are affected by proteinuria consistent with nephrotic syndrome—a possible manifestation of active pediatric LN—their role as early biomarkers and their temporal causal relationship with LN progression require validation. Moreover, whether this combination offers incremental value beyond existing clinical tools remains unclear. Prospective longitudinal studies will be essential to determine their actual predictive utility. Third, this was a single-center study with a relatively limited sample size, which may restrict the generalizability of the findings. Further validation through multicenter, large-scale prospective studies is needed. Fourth, Due to the limited number of cases of lupus nephritis included in this study, we did not perform subgroup analyses for lupus nephritis with different pathological classes (such as proliferative vs. non-proliferative nephritis). Future research should further explore the predictive value of these serological indicators across distinct pathological subtypes to establish more accurate and pathophysiology-informed prediction models for LN.

## 5 Conclusion

In conclusion, serum levels of TC, Ca, and IgG were identified as independently associated factors for lupus nephritis in cSLE. The combination of these routine laboratory parameters provides a simple and accessible strategy for discriminating prevalent LN.

## Data Availability

The raw data supporting the conclusions of this article will be made available by the authors, without undue reservation.
