# Supplementary material for: Serum total cholesterol, calcium, and IgG as discriminatory markers of lupus nephritis in children with systemic lupus erythematosus
Source: Front Pediatr. 2026 Aug 3;14:1889717. doi: 10.3389/fped.2026.1889717 (PMC13478111; doi:10.3389/fped.2026.1889717)
Supplement: Supplementary file 1 [file Table1.docx]

**Supplementary Table S1. The results of multivariate binary Logistic regression analysis**

|  | *β* | *S.E.* | *Wals* | *OR(95%CI)* | *P* value |
| --- | --- | --- | --- | --- | --- |
| Constant | 9.192 | 2.815 | 10.664 | —— | **0.001** |
| Ca (mmol/L) | -4.960 | 1.123 | 19.507 | 0.007(0.001,0.063) | **<0.001** |
| TC (mmol/L) | 0.593 | 0.135 | 19.299 | 1.809(1.388,2.356) | **<0.001** |
| FIB (g/L) | 0.321 | 0.229 | 1.959 | 1.379(0.879,2.162) | 0.162 |
| IgG (g/L) | -0.205 | 0.051 | 15.813 | 0.815(0.737,0.901) | **<0.001** |

Abbreviation: Ca: total Calcium, TC: Total cholesterol, FIB: Fibrinogen, IgG: Immunoglobulin G.
