# Supplementary material for: Serum total cholesterol, calcium, and IgG as discriminatory markers of lupus nephritis in children with systemic lupus erythematosus
Source: Front Pediatr. 2026 Aug 3;14:1889717. doi: 10.3389/fped.2026.1889717 (PMC13478111; doi:10.3389/fped.2026.1889717)
Supplement: Supplementary file 2 [file Table2.docx]

**Supplementary Table S2. Iteration history** ^a,b,c,d,e,f^

| Iteration | | -2 Log-likelihood | coefficient | | | | |
| --- | --- | --- | --- | --- | --- | --- | --- |
|  |  |  | Constant | TC | Ca | IgG | FIB |
| Step 0 | 1 | 257.303 | -0.203 |  |  |  |  |
|  | 2 | 257.303 | -0.204 |  |  |  |  |
| Step 1 | 1 | 218.131 | -2.338 | 0.405 |  |  |  |
|  | 2 | 214.113 | -3.269 | 0.588 |  |  |  |
|  | 3 | 214.008 | -3.446 | 0.624 |  |  |  |
|  | 4 | 214.008 | -3.451 | 0.625 |  |  |  |
|  | 5 | 214.008 | -3.451 | 0.625 |  |  |  |
| Step 2 | 1 | 202.688 | 2.881 | 0.391 | -2.299 |  |  |
|  | 2 | 195.899 | 3.633 | 0.613 | -3.132 |  |  |
|  | 3 | 195.587 | 3.795 | 0.675 | -3.338 |  |  |
|  | 4 | 195.586 | 3.803 | 0.679 | -3.350 |  |  |
|  | 5 | 195.586 | 3.803 | 0.679 | -3.350 |  |  |
| Step 3 | 1 | 187.485 | 5.841 | 0.302 | -2.826 | -0.120 |  |
|  | 2 | 177.033 | 8.412 | 0.523 | -4.221 | -0.169 |  |
|  | 3 | 176.112 | 9.611 | 0.606 | -4.816 | -0.192 |  |
|  | 4 | 176.101 | 9.780 | 0.615 | -4.896 | -0.195 |  |
|  | 5 | 176.101 | 9.783 | 0.616 | -4.897 | -0.195 |  |
|  | 6 | 176.101 | 9.783 | 0.616 | -4.897 | -0.195 |  |
| Step 4 | 1 | 184.002 | 5.527 | 0.277 | -2.835 | -0.126 | 0.170 |
|  | 2 | 172.892 | 7.902 | 0.495 | -4.225 | -0.176 | 0.244 |
|  | 3 | 171.739 | 9.020 | 0.581 | -4.855 | -0.201 | 0.301 |
|  | 4 | 171.715 | 9.189 | 0.592 | -4.957 | -0.204 | 0.320 |
|  | 5 | 171.715 | 9.192 | 0.593 | -4.960 | -0.205 | 0.321 |
|  | 6 | 171.715 | 9.192 | 0.593 | -4.960 | -0.205 | 0.321 |

Footnotes: a. Method: Forward stepwise (Wald); b. The model includes constants; c. Initial -2 log-likelihood: 257.303; d. The estimation terminated at iteration 2 because the change in parameter estimates was less than 0.001; e. The estimation terminated at iteration 5 because the change in parameter estimates was less than 0.001; f. The estimation terminated at iteration 6 because the change in parameter estimates was less than 0.001.

**Supplementary Table S3.** **Variables in the equation^e^**

|  | | *β* | *S.E.* | *Wals* | df | *P* value | *OR* | *95% CI* | |
| --- | --- | --- | --- | --- | --- | --- | --- | --- | --- |
|  |  |  |  |  |  |  |  | Lower Limit | Upper Limit |
| Step 0 | Constant | -0.204 | 0.147 | 1.924 | 1 | 0.165 | 0.816 |  |  |
| Step 1^a^ | TC | 0.625 | 0.116 | 29.164 | 1 | 0.000 | 1.868 | 1.489 | 2.344 |
|  | Constant | -3.451 | 0.614 | 31.616 | 1 | 0.000 | 0.032 |  |  |
| Step 2^b^ | Ca | -3.350 | 0.884 | 14.353 | 1 | 0.000 | 0.035 | 0.006 | 0.199 |
|  | TC | 0.679 | 0.126 | 29.134 | 1 | 0.000 | 1.971 | 1.541 | 2.522 |
|  | Constant | 3.803 | 1.997 | 3.627 | 1 | 0.057 | 44.832 |  |  |
| Step 3^c^ | Ca | -4.897 | 1.113 | 19.361 | 1 | 0.000 | 0.007 | 0.001 | 0.066 |
|  | TC | 0.616 | 0.132 | 21.706 | 1 | 0.000 | 1.851 | 1.428 | 2.398 |
|  | IgG | -0.195 | 0.050 | 15.026 | 1 | 0.000 | 0.823 | 0.745 | 0.908 |
|  | Constant | 9.783 | 2.784 | 12.352 | 1 | 0.000 | 17728.319 |  |  |
| Step 4^d^ | Ca | -4.960 | 1.123 | 19.507 | 1 | 0.000 | 0.007 | 0.001 | 0.063 |
|  | TC | 0.593 | 0.135 | 19.299 | 1 | 0.000 | 1.809 | 1.388 | 2.356 |
|  | FIB | 0.321 | 0.229 | 1.959 | 1 | 0.162 | 1.379 | 0.879 | 2.162 |
|  | IgG | -0.205 | 0.051 | 15.813 | 1 | 0.000 | 0.815 | 0.737 | 0.901 |
|  | Constant | 9.192 | 2.815 | 10.664 | 1 | 0.001 | 9821.399 |  |  |

Footnotes: a. Variable entered on step 1: TC; b. Variable entered on step 2: Ca; c. Variable entered on step 3: IgG; d. Variable entered on step 4: FIB; e. The stepwise process stopped because removing the least significant variable would result in a previously fitted model.

**Supplementary Table S4.** **Candidate variables not entered into the equation at each step and reasons**

| Steps | Candidate variables | Score | df | *P* value | Meets criteria for entry | Reasons for exclusion |
| --- | --- | --- | --- | --- | --- | --- |
| Step 0 | ALB/GLO | 4.100 | 1 | 0.043 | Yes | Score lower than TC |
|  | Ca | 15.888 | 1 | <0.001 | Yes | Score lower than TC |
|  | P | 4.280 | 1 | 0.039 | Yes | Score lower than TC |
|  | **TC** | **34.568** | **1** | **<0.001** | Yes (Step 1: Inclusion) | |
|  | TG | 9.883 | 1 | 0.002 | Yes | Score lower than TC |
|  | HDL | 17.361 | 1 | <0.001 | Yes | Score lower than TC |
|  | PT | 7.168 | 1 | 0.007 | Yes | Score lower than TC |
|  | APTT | 8.073 | 1 | 0.004 | Yes | Score lower than TC |
|  | FIB | 6.168 | 1 | 0.013 | Yes | Score lower than TC |
|  | IgG | 19.730 | 1 | <0.001 | Yes | Score lower than TC |
|  | IgM | 4.323 | 1 | 0.038 | Yes | Score lower than TC |
|  | C3 | 4.435 | 1 | 0.035 | Yes | Score lower than TC |
|  | Total Statistics | 74.527 | 12 | <0.001 |  |  |
| Step 1 | ALB/GLO | 0.014 | 1 | 0.905 | No | Did not meet entry criteria |
|  | **Ca** | **19.081** | **1** | **<0.001** | Yes (Step 2: Inclusion) | |
|  | P | 2.226 | 1 | 0.136 | No | Did not meet entry criteria |
|  | TG | 0.105 | 1 | 0.746 | No | Did not meet entry criteria |
|  | HDL | 0.576 | 1 | 0.448 | No | Did not meet entry criteria |
|  | PT | 0.934 | 1 | 0.334 | No | Did not meet entry criteria |
|  | APTT | 0.002 | 1 | 0.968 | No | Did not meet entry criteria |
|  | FIB | 2.587 | 1 | 0.108 | No | Did not meet entry criteria |
|  | **IgG** | **8.240** | **1** | **0.004** | Yes | Score lower than Ca |
|  | IgM | 0.284 | 1 | 0.594 | No | Did not meet entry criteria |
|  | **C3** | **4.962** | **1** | **0.026** | Yes | Score lower than Ca |
|  | Total Statistics | 49.076 | 11 | <0.001 |  |  |
| Step 2 | **ALB/GLO** | **8.597** | **1** | **0.003** | Yes | Score lower than IgG |
|  | P | 0.104 | 1 | 0.747 | No | Did not meet entry criteria |
|  | TG | 2.001 | 1 | 0.157 | No | Did not meet entry criteria |
|  | **HDL** | **4.202** | **1** | **0.040** | Yes | Score lower than IgG |
|  | PT | 1.175 | 1 | 0.278 | No | Did not meet entry criteria |
|  | APTT | 0.002 | 1 | 0.960 | No | Did not meet entry criteria |
|  | FIB | 2.192 | 1 | 0.139 | No | Did not meet entry criteria |
|  | **IgG** | **17.575** | **1** | **<0.001** | Yes (Step 3: Inclusion) | |
|  | IgM | 0.003 | 1 | 0.954 | No | Did not meet entry criteria |
|  | C3 | 0.004 | 1 | 0.947 | No | Did not meet entry criteria |
|  | Total Statistics | 33.144 | 10 | <0.001 |  |  |
| Step 3 | ALB/GLO | 0.328 | 1 | 0.567 | No | Did not meet entry criteria |
|  | P | 0.105 | 1 | 0.745 | No | Did not meet entry criteria |
|  | TG | 0.243 | 1 | 0.622 | No | Did not meet entry criteria |
|  | HDL | 1.067 | 1 | 0.302 | No | Did not meet entry criteria |
|  | **PT** | **3.967** | **1** | **0.046** | Yes | Score lower than FIB |
|  | APTT | 0.333 | 1 | 0.564 | No | Did not meet entry criteria |
|  | **FIB** | **4.533** | **1** | **0.033** | Yes (Step 4: Inclusion) | |
|  | IgM | 0.724 | 1 | 0.395 | No | Did not meet entry criteria |
|  | **C3** | **4.208** | **1** | **0.040** | Yes | Score lower than FIB |
|  | Total Statistics | 18.973 | 9 | 0.025 |  |  |
| Step 4 | ALB/GLO | 1.109 | 1 | 0.292 | No | Did not meet entry criteria |
|  | P | 0.058 | 1 | 0.810 | No | Did not meet entry criteria |
|  | TG | 0.342 | 1 | 0.558 | No | Did not meet entry criteria |
|  | HDL | 2.118 | 1 | 0.146 | No | Did not meet entry criteria |
|  | **PT** | **5.080** | **1** | **0.024** | Yes | Algorithm terminated; variable not entered |
|  | APTT | 0.183 | 1 | 0.669 | No | Did not meet entry criteria |
|  | IgM | 0.876 | 1 | 0.349 | No | Did not meet entry criteria |
|  | **C3** | **7.043** | **1** | **0.008** | Yes | Algorithm terminated; variable not entered |
|  | Total Statistics | 15.617 | 8 | 0.048 |  |  |

**Supplementary Table S5. Serum TC, Ca, and IgG Levels in LN Subgroups Stratified by ISN/RPS Class and Proliferative Status**

|  | First Analysis^a^ | | | |  | Second Analysis^b^ | | |
| --- | --- | --- | --- | --- | --- | --- | --- | --- |
| Parameters | Class I+II+III(n=28) | Class IV(n=31) | Class V(n=25) | *P* value |  | Proliferative LN (n=65) | Non‑proliferative LN (n=19) | *P* value |
| TC(mmol/L) | 5.39(4.12,6.83) | 6.23(4.96,7.37) | 6.48(4.66,7.83) | 0.238 |  | 5.80(4.55,7.13) | 6.48(5.23,8.38) | 0.085 |
| Ca(mmol/L) | 2.20(2.11,2.41) | 2.17(2.04,2.25) | 2.23(2.04,2.31) | 0.265 |  | 2.21(2.09,2.32) | 2.16(2.00,2.32) | 0.669 |
| IgG(g/L) | 9.29(7.19,12.37) | 9.38(6.11,11.10) | 8.24(5.18,11.65) | 0.544 |  | 8.85(6.11,11.55) | 9.12(5.66,10.80) | 0.962 |

Data are presented as median (interquartile range) for non‑normally distributed continuous variables. a. Differences among the three ISN/RPS classes were evaluated using the Kruskal‑Wallis test. b. Comparisons between the proliferative and non‑proliferative groups were performed using the Mann‑Whitney U test. A two‑sided *P*-value<0.05 was considered statistically significant.
